# Comparing uptake across breast, cervical and bowel screening at an individual level: a retrospective cohort study

**DOI:** 10.1038/s41416-019-0564-9

**Published:** 2019-09-04

**Authors:** Colin McCowan, Paula McSkimming, Richard Papworth, Marie Kotzur, Alex McConnachie, Sara Macdonald, Sally Wyke, Emilia Crighton, Christine Campbell, David Weller, Robert J. C. Steele, Kathryn A. Robb

**Affiliations:** 1 School of Medicine, University of St Andrews, St Andrews, KY16 9TF UK; 2 Robertson Centre for Biostatistics, Institute of Health & Wellbeing, University of Glasgow, Glasgow, G12 8QQ UK; 3 Institute of Health & Wellbeing, University of Glasgow, Glasgow, G120XH UK; 4 Institute of Health & Wellbeing, University of Glasgow, Glasgow, G129LX UK; 5 Institute of Health & Wellbeing, University of Glasgow, Glasgow, G128RS UK; 6 NHS Greater Glasgow and Clyde, Gartnavel Royal Hospital, Glasgow, G120XH UK; 7 Usher Institute, University of Edinburgh, Edinburgh, EH8 9AG UK; 8 School of Medicine, University of Dundee, Dundee, DD14HN UK

**Keywords:** Cancer screening, Population screening

## Abstract

**Background:**

We investigated demographic and clinical predictors of lower participation in bowel screening relative to breast and cervical screening.

**Methods:**

Data linkage study of routinely collected clinical data from 430,591 women registered with general practices in the Greater Glasgow & Clyde Health Board. Participation in the screening programmes was measured by attendance at breast or cervical screening or the return of a bowel screening kit.

**Results:**

72.6% of 159,993 women invited attended breast screening, 80.7% of 309,899 women invited attended cervical screening and 61.7% of 180,408 women invited completed bowel screening. Of the 68,324 women invited to participate in all three screening programmes during the study period, 52.1% participated in all three while 7.2% participated in none. Women who participated in breast (OR = 3.34 (3.21, 3.47), *p* < 0.001) or cervical (OR = 3.48 (3.32, 3.65), *p* < 0.001) were more likely to participate in bowel screening.

**Conclusion:**

Participation in bowel screening was lower than breast or cervical for this population although the same demographic factors were associated with uptake, namely lower social deprivation, increasing age, low levels of comorbidity and prior non-malignant neoplasms. As women who complete breast and cervical are more likely to also complete bowel screening, interventions at these procedures to encourage bowel screening participation should be explored.

## Background

Screening can reduce deaths from cancer if people are willing to participate. Population-based surveys in the USA and UK have found public enthusiasm for cancer screening to be around 90%,^1,2^ but uptake of screening for women in Scotland is much lower, at 72% for breast, 73% for cervical but only 60% for bowel.^3–5^ The differential between public support and participation is most obviously seen in bowel screening and a better understanding of why it fails to achieve the same uptake rates of breast and cervical screening is needed.

In Scotland, women are invited to participate in three national cancer screening programmes. For breast screening, women are invited to attend for mammogram every 3 years between the ages of 50 and 69 years. For cervical screening, since June 2016 women are invited between the ages of 25–49 every 3 years and women aged 50–64 are invited every 5 years, but prior to that women aged 20–59 years were invited to cervical screening every 3 years. For bowel screening, men and women aged 50–74 are invited to complete the home-based guaiac faecal occult blood test (FOB test) every 2 years, although this was replaced by a Faecal Immunochemical Test (FIT) in November 2017.

The lower uptake of bowel screening has not been extensively explored in relation to the higher uptake achieved for breast and cervical screening among the same women. A survey of 890 women reported whether they had ever completed breast, cervical or colorectal screening with responses of 90, 83 and 67% for those who were eligible.^6^ Social deprivation was the only common factor associated with lower participation across the three programmes and women who did breast or cervical screening were more likely to do colorectal, than those who did not. However less than half the women surveyed were eligible for colorectal screening. One survey in the UK focussed on self-reported cervical and breast screening uptake and found that 84% had ever participated in breast or cervical screening, but that 3% had never done either. The authors also reported differential uptake by markers of social deprivation for both programmes and for ethnicity for cervical screening.^7^ A more recent paper examining socio-economic inequalities across England on uptake of cervical and breast screening reported that there was still differential uptake of cervical screening by SES but not breast screening.^8^ Our study examined factors associated with actual screening uptake (self-report tends to overestimate screening participation)^7^ to provide a better understanding of the profiles of women engaging in none, some or all forms of cancer screening. A better understanding of the numbers and characteristics of women in these groups will help in informing the development of interventions to increase bowel screening uptake. Our study aimed to investigate demographic and clinical factors associated with the lower participation in bowel screening relative to breast and cervical screening using linked data.

## Methods

The study is a cross-sectional analysis of national cancer screening programme and health-related databases available for over 1.3 million residents of the Greater Glasgow area, Scotland, UK. In Scotland, universal healthcare is provided by a publicly funded system the National Health Service (NHS), and managed at a local level by regional health boards. Each patient registered with a general practice within the Greater Glasgow and Clyde (GGC) health board has a unique 10-digit identifier called the community health index number (CHI), which allows data linkage of all NHS encounters including any participation in the three cancer screening programmes. The linked data were held within the NHS Greater Glasgow & Clyde (NHSGGC) Safe Haven.

We examined records of all female patients listed in the breast, cervical and bowel screening programmes in NHSGGC for the period 1 January 2009 to 31 December 2013. Patients were identified as invited to the three cancer screening programmes if there was a record of an invitation being sent or the individual participating in the programme. We used the term ‘completed’ if people attended breast or cervical or returned the bowel screening test kit. Data on invitations and completion of each programme were linked to hospital discharge records, use of anti-depressants from the National Prescribing Information System,^9^ the presence of learning disabilities recorded in General Practice local enhanced service data, and demographic data including age, nursing home residence and socio-economic status measured by the postcode based Scottish Index of Multiple Deprivation (SIMD) quintiles.^10^ All identifiers were removed from the original data sources and replaced with a project specific pseudonymous identifier before the research team were given access to the data via the Glasgow Safe Haven secure analytical platform.

A Charlson Index of comorbidity for each individual was calculated based on diagnostic ICD-10 codes within hospital discharge records.^11^ Prior malignant neoplasms were also identified from hospital discharge records as were non-malignant neoplasm, which included non-cancerous, in situ and other groups of neoplasms.

Data received from the cancer screening programmes included information on invites to the cervical and breast programmes and test kits sent out for the bowel programme. All attendances at breast and cervical screening and return of bowel screening test kits were also reported. All of these contacts with the screening programme were dated but there was no indication of which cycle of screening each test was for or whether it was an initial invitation or test kit or a follow-on invitation. We therefore focussed on any participation in each programme within the study period as our outcome of interest as a proxy for coverage, which is defined as participation in a screening programme within a 3 year period.^8^

### Statistical analysis

Data were analysed descriptively overall and by group (completed screening and did not complete screening) with means and standard deviations for continuous outcomes and number and percentage for categorical outcomes. Group comparisons were carried out using *t*-tests for continuous data and Fisher’s Exact tests unless otherwise stated.

To identify factors that may influence whether a woman will complete cancer screening or not, multivariable logistic regression models were created with completed screening as the outcome and the following variables as predictors: age (per 10 Years); SIMD quintiles; care home resident; learning disabled; cancer diagnosis ever; Charlson Comorbidity Index Score Category (0, 1, 2+). Odds ratios for between-group differences were calculated with 95% confidence intervals (CIs) and *p*-values. The multivariable models were then extended to include interaction terms between SIMD and each of the other predictor variables as well as the interaction between completing breast and cervical screenings. Similar models were constructed for the cohort of women who were eligible for all three screenings with completed bowel as the outcome and also the additional adjustments of completed breast screening and completed cervical screening.

All statistical analyses were performed using SAS version 9.3.

## Results

Four hundred and thirty thousand five hundred and ninety one women were invited to take part in at least one of the screening programmes during 2009–2013. One hundred and sixteen thousand two hundred and twelve (72.6%) women attended for breast screening out of 159,993 invited over the period, 250,056 (80.7%) women attended cervical screening from 309,899 invited and 111,235 (61.7%) women completed bowel screening from 180,408 invited. See Table 1 for a summary of demographic characteristics by screening programme.

**Table 1:** Characteristics of all women invited to cancer screening programmes in NHS Greater Glasgow and Clyde 2009–2013

	Breast	Cervical	Bowel
Eligible population	159,993	309,899	180,408
Ever screened between 2009–2013 (%)	116,212 (72.6)	250,056 (80.7)	111,235 (61.7)
Age of women screening (%)			
<20	0	1210 (95.0)	0
20–29	0	70,820 (72.8)	0
30–39	0	59,070 (84.4)	0
40–49	85 (55.9)	66,790 (85.1)	0
50–59	65,087 (72.1)	46,759 (81.7)	57,182 (59.7)
60–69	44,105 (72.8)	5304 (96.6)	37,886 (66.7)
70+	6935 (77.3)	103 (100.0)	16,167 (58.0)
SIMD Quintiles of socio-economic deprivation (%)			
1 (most deprived)	32,727 (63.2)	87,677 (79.7)	31,800 (52.3)
2	19,288 (71.0)	42,767 (79.8)	18,269 (59.3)
3	16,700 (74.6)	36,846 (80.2)	15,601 (63.1)
4	18,276 (79.0)	33,642 (79.5)	16,832 (68.6)
5 (most affluent)	29,221 (82.4)	49,124 (84.6)	28,733 (72.7)
Care home resident	234 (39.7)	212 (68.4)	235 (21.0)
Learning disability registered	328 (52.1)	347 (34.4)	266 (39.6)
Long term anti-depressant use	22,475 (67.2)	43,558 (86.5)	21,246 (57.4)
Previous neoplasm (%)			
None	99,312 (73.3)	233,061 (80.3)	92,228 (60.9)
Non-malignant	9037 (77.6)	11,107 (87.8)	8660 (69.0)
Malignant	7863 (61.3)	5888 (85.0)	10,347 (62.6)
Charlson Index of comorbidity during study period (%)			
0	75,905 (74.1)	210,015 (79.7)	68,803 (61.4)
1–2	31392 (72.7)	34,512 (86.9)	31,722 (64.1)
3+	8915 (62.0)	5529 (84.0)	10,710 (56.9)

Of the women who participated in each screening programme, 30,371 (26.1%) attended more than one breast screening, 75,222 (30.1%) attended more than one cervical screening and 62,385 (56.1%) returned more than one bowel screening test.

Six hundred and twenty nine women were identified as having a learning disability and were invited for breast screening, with 328 (52.1%) participating. Similarly, for cervical screening, 1008 women with a learning disability were invited with 347 (34.4%) participating and for bowel screening, 672 women were sent a kit and 266 (39.6%) participated.

Five hundred and ninety women were identified as being care home residents and invited for breast screening with 234 (39.7%) participating. Similarly, for cervical screening, 310 women living in care homes were invited with 212 (68.4%) participating, and for bowel screening, 1118 women were invited with 235 (21.0%) participating.

We also examined participation in the screening programmes of women with a diagnosis of depression, where any prescription of an anti-depressant in prescribing records during the study period was taken as a proxy for a clinical diagnosis. 33,427 women on anti-depressant medication were invited for breast screening with 22,475 (67.2%) participating, 50,351 women on anti-depressant medication were invited for cervical screening with 43,558 (86.5%) participating and 37,026 women on anti-depressant medication were invited for bowel screening with 21,246 (57.4%) participating.

Multivariable logistic regression models showed that increasing age, socio-economic status, history of a prior non-malignant neoplasm and low level of comorbidity were associated with higher participation in all three screening programmes (see Table 2). Patients resident in a care home or with a learning disability were less likely to participate in all three programmes. The presence of prior malignant cancer reduced the likelihood of participation in breast and cervical but increased the likelihood for bowel screening. Higher levels of comorbidity (Charlson Index of 3 or more) reduced participation in breast and bowel screening but had no effect on cervical screening.

**Table 2:** Multivariable Logistic Regression Models for women participating in cancer screening programmes

	Breast	Cervical	Bowel
Eligible population	159,993	309,899	180,408
Age (per additional 10 years)	1.17 (1.15, 1.19)	1.26 (1.25, 1.27)	1.11 (1.09. 1.12)
Scottish Index of Multiple Deprivation Quintiles (%)			
1 (poorest)	0.38 (0.36, 0.39)	0.76 (0.74, 0.79)	0.41 (0.40, 0.42)
2	0.53 (0.51, 0.55)	0.77 (0.74, 0.79)	0.54 (0.52, 0.56)
3	0.64 (0.61, 0.66)	0.79 (0.76, 0.81)	0.64 (0.62, 0.66)
4	0.81 (0.78, 0.85)	0.74 (0.72, 0.77)	0.83 (0.80, 0.85)
5 (most affluent)	Reference Level		
Care home resident	0.28 (0.24, 0.33)	0.56 (0.44, 0.73)	0.16 (0.14, 0.19)
Learning disability registered	0.55 (0.47, 0.65)	0.12 (0.10, 0.14)	0.55 (0.47, 0.64)
Previous neoplasm (%)			
None	Reference Level		
Non-malignant	1.29 (1.23, 1.35)	1.50 (1.42, 1.59)	1.44 (1.38, 1.50)
Malignant	0.62 (0.59, 0.65)	0.90 (0.83, 0.97)	1.09 (1.05, 1.14)
Charlson Index of comorbidity (%)			
0	Reference Level		
1–2	1.05 (1.02, 1.08)	1.45 (1.41, 1.50)	1.17 (1.15, 1.20)
3+	0.75 (0.72, 0.78)	1.06 (0.99, 1.15)	0.87 (0.84, 0.90)

Sixty eight thousand three hundred and twenty four women were invited to participate in all three screening programmes: 35,595 (52.1%) participated in all three programmes at least once, and 4934 (7.2%) participated in none during the study period 2009–2013. One thousand and sixty-four women (1.6%) participated in bowel screening only, 7601 (11.1%) participated in bowel screening and also one of breast or cervical screening and 19,130 (28.0%) participated in breast and/or cervical screening but not bowel screening.

Comparing across screening programmes the absolute difference between bowel and breast and cervical was 11% (65 vs 76%) and 19% (65 vs 84%). Interestingly across several predictors of uptake (SIMD, younger age) these differences were broadly maintained within the different categories.

The characteristics of women who were eligible for all three programmes, and participated in all three, is shown in Table 3. A high proportion of women who attended breast or cervical also participated in bowel screening, at 73.5 and 71.1%, respectively, although 19,130 women (28%) participated in breast and/or cervical screening but not bowel. Older women and increasing affluence saw higher proportions of women participate in screening. Compared to those with no prior neoplasms, higher proportions of women with prior non-malignant neoplasms participated in all three programmes although women with prior malignant cancers had higher participation in cervical and bowel but lower for breast. Women with lower levels of comorbidity had lower participation in cervical and bowel screening but higher participation for breast. Women with a high level of comorbidity had lower participation across all three programmes.

**Table 3:** Characteristics of cohort eligible for all three cancer screening programmes

	Breast	Cervical	Bowel
Eligible population	68,324	68,324	68,324
Ever screened between 2009–2013 (% of eligible population)	51,798 (75.8)	57,250 (83.8)	44,260 (64.8)
Also completed bowel screening (% of breast and cervical attendees)	38,097 (73.5)	40,694 (71.1)	44,260 (100.0)
Age of attendees (%)			
50–59	45,632 (74.8)	50,176 (82.3)	38,531 (63.2)
60+	6166 (84.1)	7074 (96.5)	5729 (78.1)
SIMD Quintiles of socio-economic deprivation (%)			
1 (most deprived)	14,158 (66.6)	16,819 (79.2)	11,818 (55.6)
2	8172 (74.1)	9131 (82.8)	6865 (62.3)
3	7350 (77.1)	7993 (83.9)	6254 (65.6)
4	8070 (81.3)	8584 (86.4)	6988 (70.4)
5 (most affluent)	14,048 (84.7)	14,723 (88.8)	12,335 (74.4)
Care home resident	40 (47.6)	41 (48.8)	28 (33.3)
Learning disability registered	120 (51.3)	77 (32.9)	88 (37.6)
Long term anti-depressant use	10,341 (70.1)	12,406 (84.1)	8935 (60.6)
Previous neoplasm (%)			
None	46,490 (76.5)	51,011 (83.6)	39,280 (64.3)
Non-malignant	3245 (78.9)	3560 (86.5)	2847 (69.2)
Malignant	1863 (59.0)	2679 (84.9)	2133 (67.6)
Charlson Index of comorbidity (%)			
0	37,851 (77.4)	40,601 (83.0)	31,582 (64.6)
1–2	11,653 (74.2)	13,584 (86.4)	10,445 (66.5)
3+	2294 (62.3)	3065 (83.2)	2233 (60.6)

A multivariable logistic regression on completion of bowel screening (Table 4) showed increased participation among older women (OR = 1.79 (1.70, 1.88) per additional 10 years, *p* < 0.001), more affluent women (*p* < 0.001 see Table 4 for ratios across each category), those with previous non-malignant (OR = 1.18 (1.09, 1.27), *p* < 0.001) or malignant neoplasms (OR = 1.43 (1.30, 1.56), *p* < 0.001) compared to no previous history, and those with a low level of comorbidity (OR = 1.08 (1.03, 1.12), *p* < 0.001) compared to women with no comorbid illness. Women who participated in breast (OR = 3.34 (3.21, 3.47), *p* < 0.001) or cervical screening (OR = 3.48 (3.32, 3.65), *p* < 0.001) were also much more likely to participate in bowel screening. Women were less likely to participate in bowel screening if they were a care home resident (OR = 0.51 (0.30, 0.85), *p* = 0.010) or had high levels of comorbidity (OR = 0.89 (0.82, 0.97), *p* = 0.008).

**Table 4:** Multivariable Logistic Regression Models for cohort of women eligible for all three cancer screening programmes

	Odds ratio 95%CI	*p*-value
Eligible population, *N* = 68 234		
Age (per additional 10 years)	1.79 (1.70, 1.88)	<0.001
SIMD Quintiles of socio-economic deprivation (%)		
1 (most deprived)	0.56 (0.53, 0.58)	<0.001
2	0.67 (0.63, 0.71)	<0.001
3	0.75 (0.70, 0.79)	<0.001
4	0.87 (0.82, 0.92)	
5 (most affluent)	Reference Level	
Care home resident	0.50 (0.30, 0.84)	0.008
Previous cancer (%)		
None	Reference Level	
Non-malignant	1.18 (1.09, 1.27)	<0.001
Malignant	1.43 (1.30, 1.56)	<0.001
Charlson Index of comorbidity (%)		
0	Reference Level	
1–2	1.08 (1.03, 1.12)	<0.001
3+	0.89 (0.82, 0.97)	0.008
Attended breast screening	3.34 (3.21, 3.47)	<0.001
Attended cervical screening	3.48 (3.32, 3.65)	<0.001

A further model examined interactions between SIMD, patient characteristics and their association with participation in cancer screening. There were significant interactions with age (*p* = 0.002), learning disabled status (*p* = 0.008) and whether the woman attended cervical screening (*p* = 0.001). For all three, the odds of participating in bowel screening were increased if a woman lived in a more affluent area. The interaction between participating in breast and cervical screening was also significant (*p* < 0.001) indicating that whether a woman attends one or both of these screenings increased their odds of attending bowel screening as well (data not reported).

## Discussion

Our findings confirm that participation in bowel screening was lower than for the breast or cervical screening programmes in women eligible for all three. Our results also highlight that while 93% participate in at least one programme and 52% participate in all three, 7% of women do not participate in any of the programmes.

The impact of demographic, clinical and socio-economic factors on participation in all programmes was similar. Lower participation associated with increased deprivation followed known patterns,^8,12^ although the association seemed less pronounced for cervical screening. Differences we report could be in part due to the dynamics of screening modalities where women may find it easier to participate in a “passive” screening procedure (i.e. having a mammogram) rather than self-completion (e.g. FOBT at home or HPV self-sampling). Our data also relate well to the self-reported data from Lo et al.^6^ that women are less likely to participate in bowel than breast or cervical. We did not directly compare participation in breast vs cervical as our focus was on bowel, but found 7% who did no screening compared to the self-reported figure of 3% for only these two programmes.^7^ Our analysis showed women with learning disabilities, resident in care or nursing homes, have much lower levels of participation. Women with depression, identified by use of anti-depressants, had lower levels of participation in bowel and breast but not cervical screening. A further, more detailed analysis of the impact of individual morbidity and multi-morbidity on participation rates could provide more information.

We believe this is the first study to report on participation across three separate cancer screening programmes for women at an individual level for a large geographically defined population. We examined the entire population in Glasgow eligible for screening and although Glasgow has higher proportions of women living in deprived areas than the rest of Scotland we know of no reason why women’s attitudes to screening may be different in Glasgow to elsewhere. We were unable to match invitation and attendances to cycles of each programme, which may reflect our reporting of slightly higher participation in each of the screening programmes than published data for Scotland. This also meant eligibility for the programmes was based on which women were invited or completed screening, which included women outside the recommended age criteria for each programme, although this was only 2% of women attending for cervical screening and 4% for breast screening.

We have conducted exploratory qualitative interviews with women with different screening histories. These data show that women who participated in breast and cervical screening but not colorectal cancer screening, were more worried about outcomes from colorectal than breast or cervical cancer, that they thought there was less need to screen for colorectal cancer as it could be picked up symptomatically more easily than breast or cervical cancer, they were concerned about how to do the actual test, and that it was easier to forget or delay. A detailed presentation of these data is currently under review.

Our data suggest that women are more likely to complete bowel screening if they participate in another screening programme. Although from this quantitative study it is not clear why bowel screening uptake should be lower, breast and cervical screening programmes could be used as a vehicle to promote participation in bowel screening. It should be noted that there was a core of women who did not participate in any programme and it will be important to establish if this reflects informed choice or a failure of current screening invitation strategies to engage with this group. Our data also suggest that women with learning disabilities or who are resident in care homes are much less likely to participate in all screening programmes and clinical care teams should think how best to ensure screening is undertaken in these populations where appropriate.

## Data Availability

The data used within this project were provided under a Local Privacy Advisory Committee approval to use a Safe Haven setting, which means the data can only be used for the specific purposes of this study and cannot be shared. However, third parties interested in making use of this data can make a separate application to LPAC and the Glasgow Safe Haven for permission to reuse this data for subsequent studies.
